# Comparison of the results of laparoscopic appendectomies with application of different techniques for closure of the appendicular stump

**DOI:** 10.1186/s13017-015-0060-3

**Published:** 2016-01-06

**Authors:** Marcin Strzałka, Maciej Matyja, Kazimierz Rembiasz

**Affiliations:** 2nd Department of General Surgery, Jagiellonian University, Kraków, Poland

**Keywords:** Laparoscopic appendectomy, Results, Outcome, Techniques, Titanium clips, Staplers, Purse string suture

## Abstract

**Background:**

Nowadays laparoscopy is used frequently not only in elective surgery but also in abdominal emergencies, including acute appendicitis. There are several techniques used to close the appendicular stump during laparoscopic appendectomy. The aim of the study was to present and compare the results of minimally invasive appendectomies performed with the use of endoscopic staplers (group A), titanium endoclips (group B) and invaginating sutures (group C).

**Methods:**

Three hundred seven patients (mean age = 35.6; SD = 15.9; 178 males,129 females) operated on laparoscopically for acute appendicitis from January 2010 to December 2014 at our department were included in the study. We reviewed retrospectively patients’ data including: age, sex, duration of the surgical procedure and hospital stay, mortality, intraoperative and postoperative complication rates in all analyzed groups.

**Results:**

There were 102 patients in group A (mean age = 35.8;SD = 15.4; 57 males, 45 females). The average hospital stay in this group was 4.3 days (SD = 1.7), average operation time was 62.0 min (SD = 15), postoperative complication rate was 5.9 %. There were 160 patients in group B (mean age = 35.0; SD = 16.3; 96 males, 64 females). The average hospital stay in this group was 3.6 days (SD = 1.4), average operation time was 62.9 min (SD = 13.5), postoperative complication rate was 5.6 %. There were 45 patients in group C (mean age =37.3; SD = 15.8; 25 males, 20 females). The average hospital stay in this group was 4.6 days (SD = 2.0), average operation time was 73.9 min (SD = 20.8), postoperative complication rate was 6.7 %. There were no intraoperative complications and no mortality in all compared groups of patients operated on laparoscopically for acute appendicitis.

**Conclusions:**

Laparoscopic appendectomies with application of different techniques for closure of the appendicular stump are useful and safe. In our study the shortest hospital stay and lowest complication rate were observed in patients operated with the use of titanium endoclips. The longest hospital stay and operation time and the highest complication rate was associated with the use of invaginating sutures.

## Background

Nowadays laparoscopy is used frequently not only in elective surgery but also in abdominal emergencies, including acute appendicitis [1–3]. Laparoscopic appendectomy is currently a well-established and widely accepted method [3, 4]. It has a lot of advantages as compared with open approach technique, including less pain in the postoperative period, faster return to normal activity and work, shorter hospital stay and lower percentage of wound infections. However some authors point out a slightly higher rate of intra-abdominal abscesses and higher cost of laparoscopic procedure as compared with the open approach operation [5, 6]. It seems that both of these factors unfavorable for laparoscopy are influenced by the applied method of the appendicular stump closure.

There are several techniques used to close the appendicular stump during laparoscopic appendectomy. The most commonly used surgical methods are connected with the use of endo-loop ligature, laparoscopic staplers, metal or polymer clips or application of purse string suture with the invagination of the appendicular base into the cecum, as in the classic surgery [7–12]. However, the optimal technique of the appendicular stump closure still seems to be controversial.

The aim of the study was to present and compare the results of minimally invasive appendectomies performed with the use of endoscopic staplers (group A), titanium endoclips (group B) and purse string invaginating sutures (group C).

## Methods

Three hundred seven patients (mean age = 35.6; SD = 15.9; 178 males, 129 females) who underwent laparoscopic appendectomies from 2010 to 2014 at our department were included in the study. Depending on the appendicular stump closure technique our patients were divided into 3 subgroups: A (endoscopic staplers), B (titanium endoclips), C (purse string invaginating sutures).

We reviewed retrospectively patients’ data including: age, sex, duration of the surgical procedure and hospital stay, mortality, intraoperative and postoperative complication rates in all analyzed groups.

**Fig. 1:**
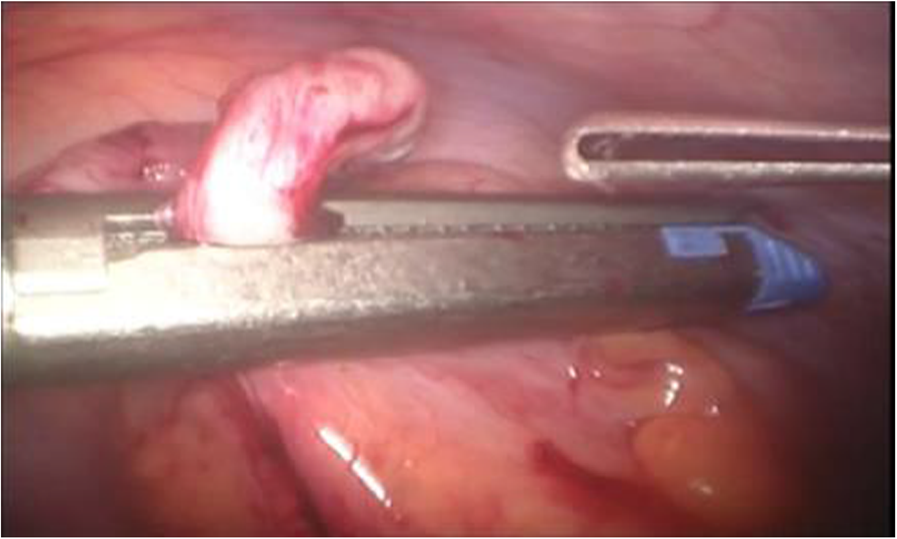
Closing of the appendicular stump with the application of laparoscopic stapler

**Fig. 2:**
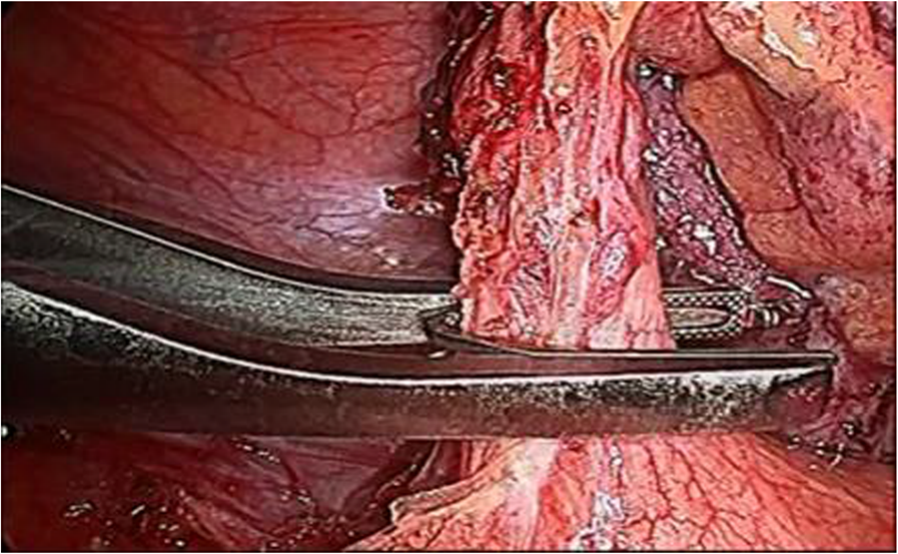
Closing of the appendicular stump with the application of titanium endoclip

**Fig. 3:**
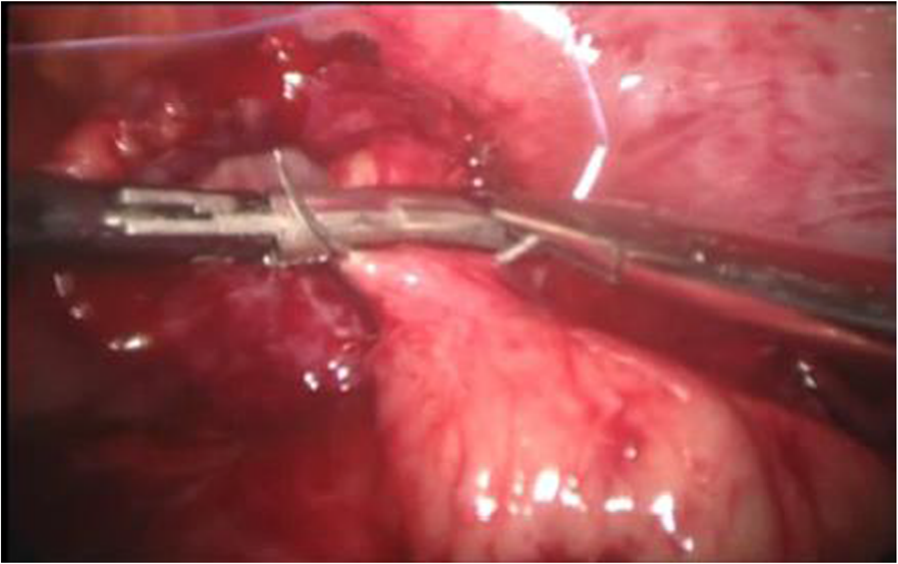
Closing of the appendicular stump with the application of purse string invaginating suture

Laparoscopic appendectomy was performed using classic three port technique. During the procedure the patient was lying on his/her back, the operating and assisting surgeons were standing at the left side and the laparoscopy unit with the monitor were placed at the right side of the patient. The majority of the procedures were performed by residents assisted by qualified surgeons since our institution is a teaching hospital. The minority of laparoscopic appendectomies were performed by specialists. Pneumoperitoneum was created using an open technique. The first trocar, through which the laparoscope was introduced, with the diameter of 10 or 11 mm, was placed in the umbilicus. The second, 5 mm port was localized in the right mesogastric area. The third trocar with a diameter of 10 or 13 mm was inserted in an oblique, not perpendicular way through a skin incision localized in the hypogastric region a little bit to the left from the middle line, and then through the fascia in the middle line and through the parietal peritoneum a little bit to the right from the middle line. Then, the patient was positioned in the Trendelenburg with a mild left tilt, to facilitate the exposure of the right lower quadrant. After the inspection of the abdominal cavity organs and confirming the diagnosis of acute appendicitis, appendix was mobilized. Its mesoappendix was usually cut with the application of electrocautery and only in few cases with the use of harmonic scalpel. The methods of the appendix stump closure differed in three analized groups. In patient group A we used in this purpose laparoscopic staplers of endo-GIA type (Fig. 1). In group B the base of the appendix was closed with the application of the metal clips with a special reusable clipper. Aesculap titanium clips were used, having a special closing mechanism at the end, protecting against slipping out of the clipped tissue (Fig. 2). Then the appendix was cut off with the electrocoutery. After the appendix section, the extraverted appendicular mucosa was coagulated. In group C the appendicular stump closure was associated with the use of the purse string suture with the invagination of the appendicular base into the cecum, as in the open surgery, without the use of any other suture or endo-loop (Fig. 3). The abdominal cavity was irrigated with warm saline solution and suctioned dry under direct visualization. In all patient groups the resected appendix was removed from the abdominal cavity in a retrieval bag through the port localized in the hypogastric area and sent for histopathological study. A tube descending into the pelvis was routinely used. It was inserted through the 5 mm trocar placed in the right mesogastric region.

We used the intravenous antibiotic prophylaxis in form of a single dose of: cefazolin (1 g in patients weighing less than 80 kg or 2 g in weighing more than 80 kg, 30 min before the procedure), and metronidazol (15 mg/kg of body weight, 60 min before the surgery).

Depending on the severity of inflammation found intraoperatively intravenous antibiotic therapy was used. It usually consisted of the third generation of cephalosporins (ceftriaxone) and metronidazol or clindamycin (for anaerobic bacteria).

We used the non-parametric Kruskal-Wallis test, post hoc tests and chi 2 test in statistical analysis of our study results.

## Results

There were 102 patients in group A, including 57 males (56 %) and 45 females (44 %). The mean patient age was 35.8 years (SD = 15.4; range:18–86 years). The average hospital stay in this group was 4.3 days (SD = 1.7), the mean operation time was 62.0 min (SD = 15), complication rate was 5.9 % (6 cases: 2 relaparosopies because of the bleeding, 1 diarrhea, 1 prolonged drainage, 1 wound infection, 1 exacerbation of IHD).

There were 160 patients in group B, including 96 males (60 %) and 64 females (40 %). The mean patient age was 35.0 years (SD = 16.3; range:18–86 years). The average hospital stay in this group was 3.6 days (SD = 1.4), the mean operation time was 62.9 min (SD = 13.5), complication rate was 5.6 % (9 cases: 1 relaparoscopy bacause of ileus caused by adhesions, 1 hematoma, 1 wound infection, 3 cases of prolonged drainage, 1 allergic rash caused by clindamycin, 2 cases of exacerbation of IHD).

There were 45 patients in group C, including 25 males (56 %) and 20 females (44 %). The mean patient age was 37.3 years (SD = 15.8; range:18–87 years). The average hospital stay in this group was 4.6 days (SD = 2.0), the mean operation time was 73.9 min (SD = 20.8), complication rate was 6.7 % (3 cases: 1 relaparoscopy because of the bleeding, 1 wound infection, 1 prolonged drainage).

We observed no intraoperative complications in our material. There was also no mortality in all compared groups.

The non-parametric Kruskal-Wallis test and post hoc tests were used for statistical analysis of variances in order to compare the results in 3 patient groups.

**Fig. 4:**
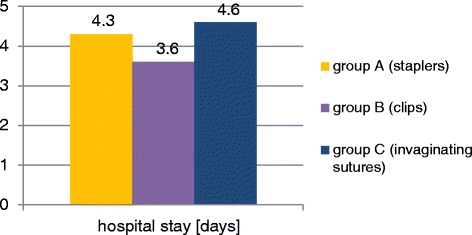
The average hospital stay in the compared groups

They showed that there are significant differences in hospital stay between groups: A (staplers) and B (clips): *p* = 0.0049 and also B (clips) and C (invaginating sutures): *p* = 0.0227 (Fig. 4, Table 1).

**Table 1:** Statistical analysis of the hospital stay

Group	*p* value for compared variables; hospital stay:		
	Kruskal-Wallis test: H ( 2, N = 307) =13.87762 *p* = .0010		
	A (staplers)	B (clips)	C (invaginating sutures)
	R:171.79	R:136.37	R:176.38
A (staplers)		0.004906	1.000000
B (clips)	0.004906		0.022665
C (invaginating sutures)	1.000000	0.022665	

**Fig. 5:**
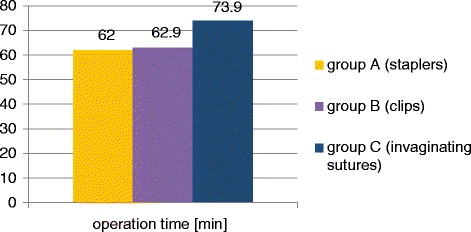
The average operation time in the compared groups

The non-parametric Kruskal-Wallis test and post hoc tests showed that there are significant differences in operation time between groups: B (clips) and C (invaginating sutures): *p* = 0.00012 and also A (staplers) and C (invaginating sutures): *p* = 0.000115 (Fig. 5, Table 2).

**Table 2:** Statistical analysis of the operation time

Group	*p* value for compared variables; operation time:		
	Kruskal-Wallis test: H(2,N = 307) = 20.05842 *p* = .0000		
	A (staplers)	B (clips)	C (invaginating sutures)
	R:141.93	R:145.75	R:207.12
A (staplers)		1.000000	0.000115
B (clips)	1.000000		0.000120
C (invaginating sutures)	0.000115	0.000120	

**Fig. 6:**
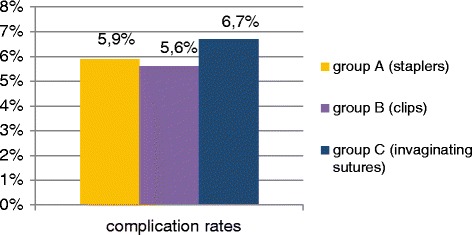
The complication rates in the compared groups

The Chi 2 test showed that there is no statistically significant correlation between the compared 3 groups and the postoperative complication rates (*p* = 0.3192) (Fig. 6).

**Fig. 7:**
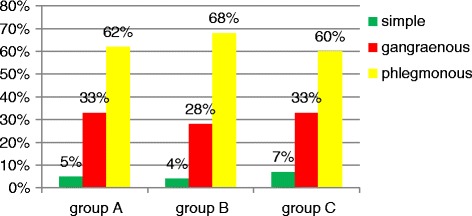
Types of appendicitis in the compared groups

No statistically significant differences were detected between the groups in terms of the distribution of age and sex percentage (*p* > 0.05). All groups were also similar when compared according to the type of appendicitis (Fig. 7).

## Discussion

The proper closure of the appendicular stump during laparoscopic appendectomy is very important because it can prevent the occurrence of a number of serious postoperative complications. Endo-loops or laparoscopic staplers of endo-GIA type are frequently used to close the appendicular stump in case of minimally invasive appendectomy [7, 8]. According to some authors, this last method is the safest option, but at the same time the most expensive, what nowadays plays an important role in many countries [8]. On the other hand, appendicular stump closure during laparoscopic appendectomy with the application of purse string suture with its invagination into the cecum, although safe and cost-effective, is much more technically demanding and requires some experience in laparoscopic sewing [9].

In recent years laparoscopic clips were suggested as an alternative method of the appendicular stump closure during minimally invasive operation of the appendix removal. Several reports presented the results of the application of non-absorbable polymeric clips Hem-o-lok, which proved to be safe, easy to use and relatively cheap [10, 11]. However, their safe use is significantly limited by the maximum diameter of the closing appendix of 10 mm. The inflamed appendix is often thicker, what makes their application impossible.

The solution of this problem can be associated with the use of metal clips [12–16]. In our study we presented the results of laparoscopic appendectomies performed with the application of metal clips made of titanium with double “jaws” and a special locking mechanism at the end preventing from slipping out the clipped tissue and coming off the clip. They allow for the closure of appendices with a diameter of 20 mm. Clipper used to apply this type of endoclips requires the use of a 12.5 or 13 mm trocar. The same trocar diameter is necessary in case of the laparoscopic staplers application. It is slightly greater than in case of the plastic clips used to close the appendicular stump, when it is possible to use the port diameter of 10 mm.

In our material the average duration of the laparoscopic appendectomy in group A and B was very similar (60.0 min and 60.9 min). The longest operation time was observed in patient group C (73.9 min), in which the purse-string suture was used. It was caused by the fact that this method of the appendicular stump closure seems to be the most technically demanding. Nevertheless, the mean durations of the surgical procedures observed in all three groups were comparable to the literature data, especially taking into account that many operations were performed by residents with assistance of specialists [12, 15].

In our study the average hospital stay was the shortest in group B. It was longer in group A and C. These differences were statistically significant and could be at least partially caused by the differences in the postoperative complication rates. The mean postoperative hospital stay in all patient groups did not differ from data presented in other publications [3, 14].

We observed no intraoperative complications and no mortality in all three compared patient groups. The postoperative complication rates were small and they did not differ statistically significantly in all analyzed patient groups. The postoperative complication rates observed in our study were also similar to other reports [9, 12, 13, 16–18]. The small percentage of surgical site infections indicate that all used techniques for appendicular stump closure are safe. In our opinion just few surgical wound infections and no intra-abdominal abscess formation in our patients is associated with the intraoperative suction and lavage of the peritoneal cavity, routinely used drains and the proper administration of antibiotics. Prolonged postoperative drainage which occurred in some patients with gangraenous appendicitis and perforation caused only prolongation of their hospital stay. However, routinely used drainage probably secured these patients from intra-abdominal abscesses formation and possible need for next surgical intervention.

Data from literature and our results indicate that the management of appendicular stump with application of metal clips during laparoscopic appendectomy appears to be simple, safe, and a cost-effective alternative [12–16, 19]. It is associated with low complication rates even in cases of complicated acute appendicitis [16]. Because of these advantages surgical technique connected with titanium clips is now the most frequently used method of laparoscopic appendectomy in our department. However, in cases of necrosis or very large thickening of the inflamed appendicular base using of the alternative method should be considered. All three techniques of the appendicular stump closure can be safely and effectively used also in case of a single incision laparoscopic approach for appendectomy [20].

## Conclusions

Laparoscopic appendectomies with application of different techniques for closure of the appendicular stump are useful and safe. In our study the shortest hospital stay and lowest complication rate were observed in patients operated on with the use of laparoscopic clips. However, the complication rate differences were statistically insignificant in all analyzed groups. The longest time of surgical procedure was connected with the application of the purse–string invaginating sutures, which is the most technically demanding, but also the cheapest method used for appendicular stump closure.
